# Rutting and Fatigue Cracking Resistance of Waste Cooking Oil Modified Trinidad Asphaltic Materials

**DOI:** 10.1155/2015/385013

**Published:** 2015-08-02

**Authors:** Rean Maharaj, Vitra Ramjattan-Harry, Nazim Mohamed

**Affiliations:** The University of Trinidad and Tobago, Point Lisas Industrial Estate, Couva, Trinidad and Tobago

## Abstract

The influence of waste cooking oil (WCO) on the performance characteristics of asphaltic materials indigenous to Trinidad, namely, Trinidad Lake Asphalt (TLA), Trinidad Petroleum Bitumen (TPB), and TLA : TPB (50 : 50) blend, was investigated to deduce the applicability of the WCO as a performance enhancer for the base asphalt. The rheological properties of complex modulus (*G* ^*∗*^) and phase angle (*δ*) were measured for modified base asphalt blends containing up to 10% WCO. The results of rheology studies demonstrated that the incremental addition of WCO to the three parent binders resulted in incremental decreases in the rutting resistance (decrease in *G* ^*∗*^/sin*δ* values) and increases in the fatigue cracking resistance (decrease in *G* ^*∗*^sin*δ* value). The fatigue cracking resistance and rutting resistance for the TLA : TPB (50 : 50) blends were between those of the blends containing pure TLA and TPB. As operating temperature increased, an increase in the resistance to fatigue cracking and a decrease in the rutting resistance were observed for all of the WCO modified asphaltic blends. This study demonstrated the capability to create customized asphalt-WCO blends to suit special applications and highlights the potential for WCO to be used as an environmentally attractive option for improving the use of Trinidad asphaltic materials.

## 1. Introduction

Asphalt is the binder used in conjunction with mineral aggregate such as limestone and gravel to construct road pavements. The inevitable aging of road pavements due to years of exposure to traffic loads and climatic and environmental changes ultimately results in the reduction in the performance of the asphalt binder and consequentially the performance of the pavement. In order to restore or even produce better-performing asphaltic binder materials to meet performance standards, expired pavement surfaces can be milled and recycled [1, 2]. Another strategy employed is the production of polymer modified asphalt binders which have the added advantage of reducing the occurrence of rutting and fatigue cracking [3].

The rutting process can be explained as the permanent deformation of asphaltic based pavements whereas fatigue cracking occurs as the binder ages losing resilience due to volatilization of smaller molecules or oxidation of organic functional groups that results in the pavement becoming brittle [4]. Since fatigue cracking usually occurs in the early life of an asphaltic pavement, it can be a precursor for rutting as the cracks that develop render the exposed areas susceptible to moisture and oxygen exposure that can accelerate the rutting process [5]. It has been reported that polymer modified asphalt can increase the life of the pavement by up to 10 years [6, 7].

Asphalt binders are considered viscoelastic; they behave partly like an elastic solid (recoverable deformation after loading) and partly like a viscous liquid (nonrecoverable deformation after loading). Since the dynamic (oscillatory) shear rheology (DSR) testing technique is capable of quantifying both elastic and viscous properties and in particular the measured rheological values of complex modulus (*G* ^*∗*^) and phase angle (*δ*), it has been recommended for the characterization of the viscoelastic properties of asphaltic material [8]. Mathematical correlations between rheological parameters (*G* ^*∗*^ and *δ*) and pavement performance attributes such as rutting and fatigue cracking have been presented by the Strategic Highway Research Program: Asphalt Research Program [9]. They suggested that as asphaltic pavements are deformed due to traffic loads, some degree of recovery occurs and energy is recovered (elastic property) while some energy is dissipated in the form of permanent deformation (rutting) and fatigue cracking. Thus to minimize deformation (rutting) and fatigue cracking, the work dissipated per load cycle (*W*_*c*_) must be minimized. *W*_*c*_ at a constant stress (*W*_*c*1_) can be expressed as shown in

(1) $$\begin{array}{c} W_{c1}=\pi \sigma_{o}^{2}\frac{1}{G^{*}/\sin\delta }, \end{array}$$

where *σ*_*o*_ is the stress applied during the load cycle. Equation (1) shows that in order to minimize rutting deformation *G* ^*∗*^/sin⁡*δ* should be increased.

*W*_*c*2_ is the work dissipated per load cycle at a constant strain and can be expressed as shown in

(2) $$\begin{array}{c} W_{c2}=\pi \varepsilon_{o}^{2}\left(\;G^{*}\sin\delta \;\right), \end{array}$$

where *ε*_*o*_ is the strain during load cycle. Equation (2) shows that in order to minimize fatigue cracking *G* ^*∗*^sin⁡*δ* should be minimized.

Apart from the Strategic Highway Research Program's utilization of the rheology-performance relationship described above, the Asphalt Research Program Superpave specification has also utilized the relationship recommending a high *G* ^*∗*^ (stiffness) but low *δ* (elastic) structure to reduce rutting and low values of *G* ^*∗*^ and *δ* to reduce the fatigue cracking [10].

The issue of the disposal of waste cooking oil (WCO), a product of the frying and cooking activities at high temperatures generated mainly from the food industry, restaurants, hotels, and residences, has become a major environmental issue [11–13]. With the concern of high construction cost and natural resource conservation, waste oil recycling is becoming a viable alternative in mitigating these problems [12]. The blending of recycled asphalt with WCO has been shown to improve the performance qualities of the resulting blends as the fatty acids present in WCO act as cohesive agents, reducing the high viscosity of the aged, recycled binders and facilitating homogenous mixing when integrated with new pavement materials [14]. The reduction in viscosity of the resulting binder has the added advantage as it results in a consequential reduction in the surface tension between the aggregate and binder coating, expelling entrapped air and increasing interfacial cohesion between the asphaltic binder and aggregate, thus improving its mechanical properties.

Of key importance is the observation in previous studies of a clear relationship between the differences in the quality of asphalt (different compositions) from different sources and the resulting performance qualities of the binders [15–19]; asphaltic materials with the same specifications can often produce pavements of varying physical properties, performance, and serviceability. Consequently bitumen and asphalt materials may interact with polymeric additives differently and rheological performances qualities, such as rutting and fatigue cracking resistance, are very dependent on the source of asphalt/bitumen used due to the varying chemical composition. Thus, the influence of additives on the rheological properties of bituminous materials cannot be easily generalized. Previous studies measuring the influence of WCO on the rheological properties of rutting resistance and fatigue cracking on the bituminous materials, TLA and TPB, indigenous to Trinidad have proven to be limited. The most famous natural deposit of asphalt is the Trinidad Lake Asphalt (TLA), which exists in a deposit on the island of Trinidad, and it is also one of chief commercial importance. The material comprises a unique mixture of bitumen (63%) and mineral matter which is kaolinitic in nature [19]. TLA has been internationally well established as a commercial product and a source of superior quality asphalt (due to its consistent properties, resistance to cracking, stability, and durability) and is often specified as a mandatory ingredient for paving in high demand situations such as those encountered on airport runways [20]. Typically, a 50 : 50 blend of TLA and refinery asphalt such as TPB is adopted in the production of TLA modified mastic.

The purpose of this study is therefore to investigate the influence of WCO on fatigue cracking resistance and rutting resistance of TLA and TPB modified blends and hence to evaluate its potential reuse in asphalt pavement applications.

## 2. Method and Materials

The TLA and TPB asphalt binders used in this study were obtained from the Lake Asphalt Company of Trinidad and Tobago and the Petroleum Company of Trinidad and Tobago Limited, respectively. The 50 : 50 TLA/TPB mix was prepared by mixing equal amounts of TLA and TPB. A gallon of WCO was obtained from a commercial restaurant in South Trinidad.

### 2.1. Sample Preparation

The sample blends were prepared using the recommended process [21]. Aluminium cans of approximately 500 cm^3^ were filled with 250 to 260 g of the asphalt binder and put in a thermoelectric heater Thermo Scientific Precision (Model 6555) where the temperature was raised to 200°C. A digital IKA (Model RW20D) high shear mixer was then immersed in the can and set to 3000 rpm. The WCO was added (by weight %) gradually while the system was kept at a temperature of 200 ± 1°C.  Table 1 documents the concentrations used in this study.

At the end of mixing, each blend was stored in a desiccator under static conditions and in an oxygen-free environment. After 24 hours of curing, the cans were taken out and remixed using the high shear mixer, and the molten mixtures were then cast into a ring stamp of 25 mm diameter and 1 mm thickness for subsequent rheological testing. Before testing, the samples were cooled at room temperature and stored in a Fisher Isotemp freezer at −20°C.

### 2.2. Sample Characterization

The rheological properties of the asphaltic materials were determined using an ATS RheoSystems Dynamic Shear Rheometer (Viscoanalyzer DSR). The analyses were performed under the strain-control mode and the applied strain was kept low enough to ensure that all the analyses were performed within the linear viscoelastic range. The test geometry used was the plate-plate configuration (diameters 25 mm) with a 1 mm gap and the measurements were conducted at the temperatures 40, 50, 60, 70, 80, and 90°C for TLA and TPB and its blends and a frequency range of 0.1–15.91 Hz. The data obtained at different oscillating shear frequencies and temperatures were stored in the computer and the results obtained were analyzed using the Viscoanalyzer software. The values of the rheological parameters associated with the complex modulus (*G* ^*∗*^) and phase angle (*δ*) were calculated at the different oscillating frequencies and temperatures using the instrument's software.

## 3. Results and Discussion

Rheological measurements of the complex modulus (*G* ^*∗*^) and phase angle (*δ*) of three Trinidad asphaltic binders TLA, TPB, and TLA : TPB (50 : 50) blend containing varying amounts of WCO were measured. The mathematical correlations between the rheological parameters *G* ^*∗*^ and *δ* and the pavement performance characteristics of rutting and fatigue cracking as outlined by the Strategic Highway Research Program [9] were utilized.

**Figure 1:**
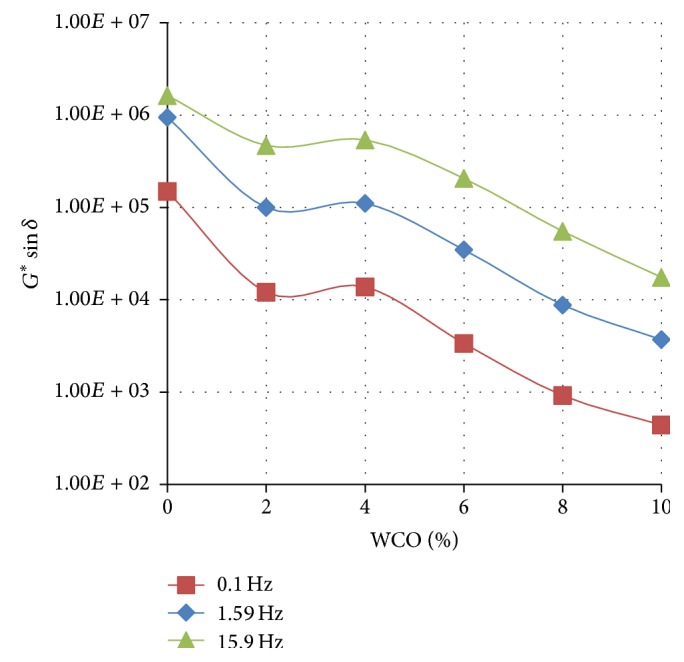
The variation of the fatigue cracking resistance parameter (*G* ^*∗*^sin⁡*δ*) with increasing concentration of WCO in TLA at various oscillating frequencies at 60°C.

**Figure 2 fig2:**
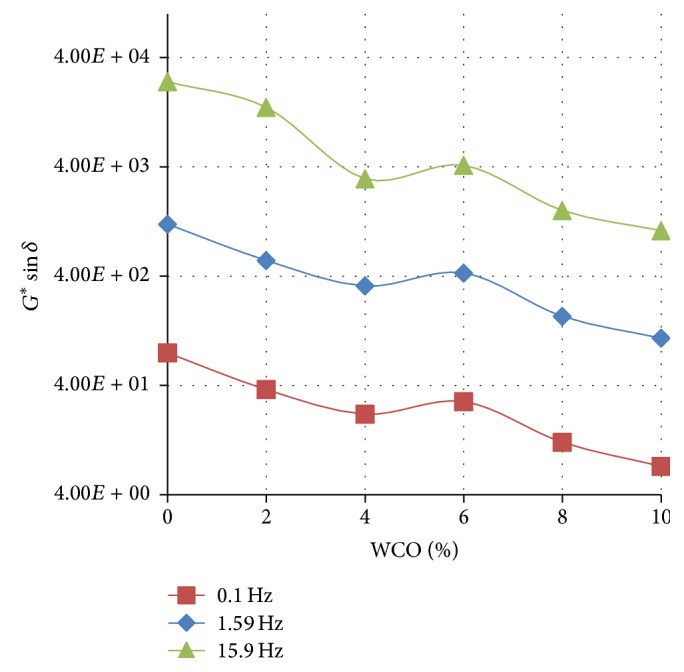
The variation of the fatigue cracking resistance parameter (*G* ^*∗*^sin⁡*δ*) with increasing concentration of WCO in TPB at various oscillating frequencies at 60°C.

**Figure 3:**
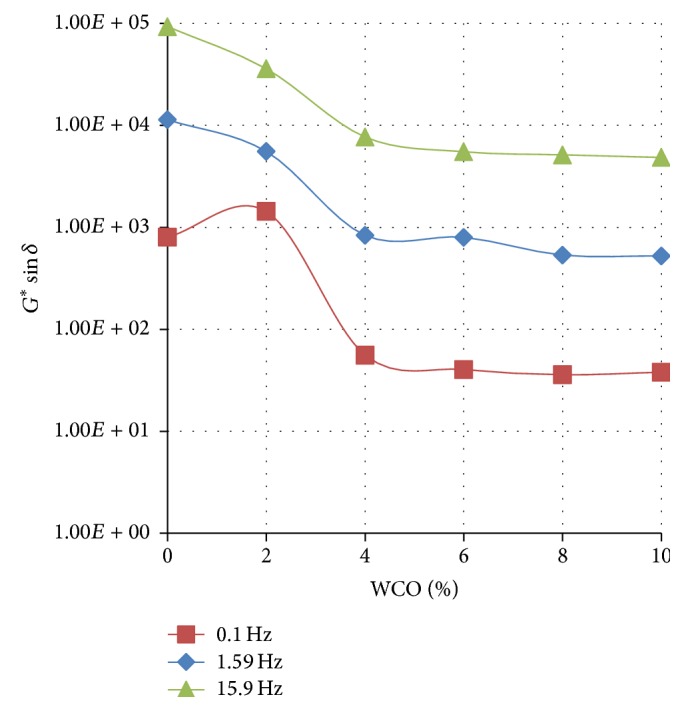
The variation of the fatigue cracking resistance parameter (*G* ^*∗*^sin⁡*δ*) with increasing concentration of WCO in TLA : TPB (50 : 50) at various oscillating frequencies at 60°C.

Figures 1 [Fig fig2]–3 show the variation of the fatigue cracking resistance parameter (*G* ^*∗*^sin⁡*δ*) with increasing concentration of WCO in TLA, TPB, and TLA : TPB (50 : 50) blend at three oscillating frequencies, respectively.

The results clearly show that for all three asphaltic binders as the concentration of WCO increases, the fatigue cracking resistance increases (*G* ^*∗*^sin⁡*δ* value decreases). As shown in Figure 3, the blends formulated using the TPB binder had the lowest values of *G* ^*∗*^sin⁡*δ* at the measured frequencies indicating that these blends will exhibit the maximum fatigue cracking resistance of all the binders used. It is interesting to note that the values of *G* ^*∗*^sin⁡*δ* at the measured frequencies for the TLA : TPB (50 : 50) blends were between those of the blends containing TLA and TPB alone, an expected observation as the TLA : TPB (50 : 50) blends are mixtures of TLA and TPB.

**Figure 4:**
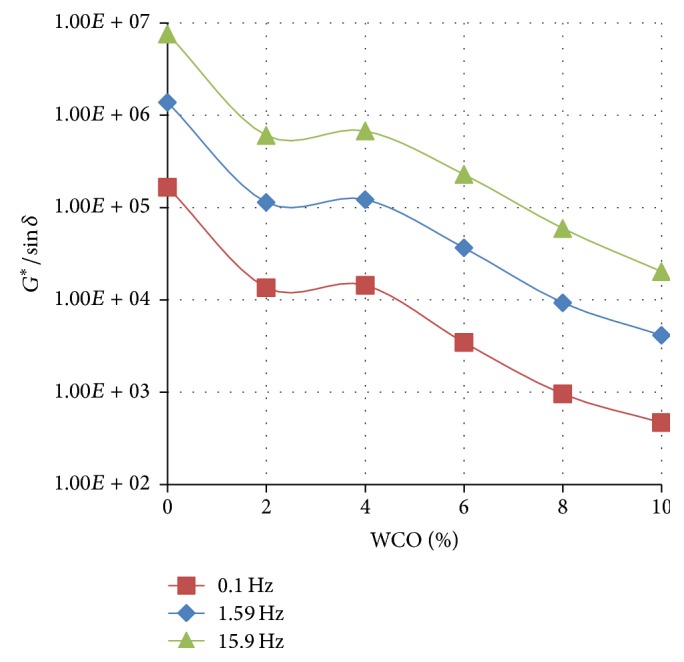
The variation of the rutting resistance parameter (*G* ^*∗*^/sin⁡*δ*) with increasing concentration of WCO in TLA at various oscillating frequencies at 60°C.

**Figure 5 fig5:**
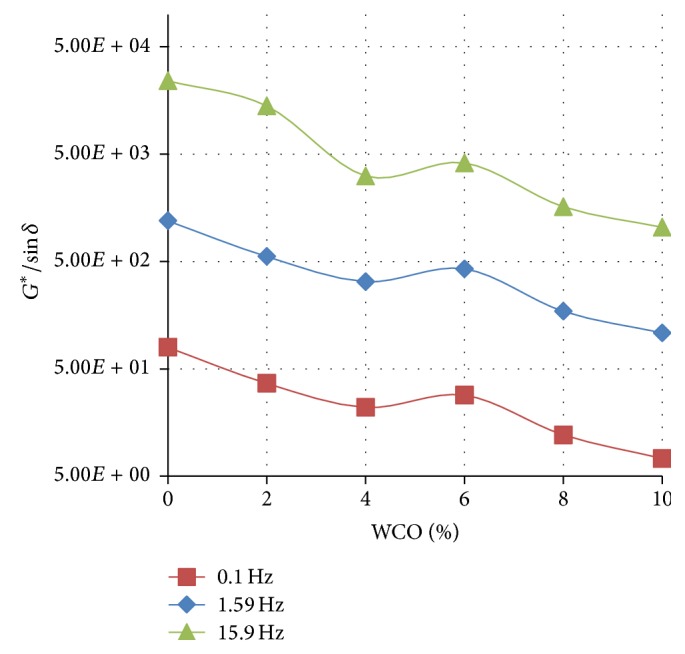
The variation of the rutting resistance parameter (*G* ^*∗*^/sin⁡*δ*) with increasing concentration of WCO in TPB at various oscillating frequencies at 60°C.

**Figure 6:**
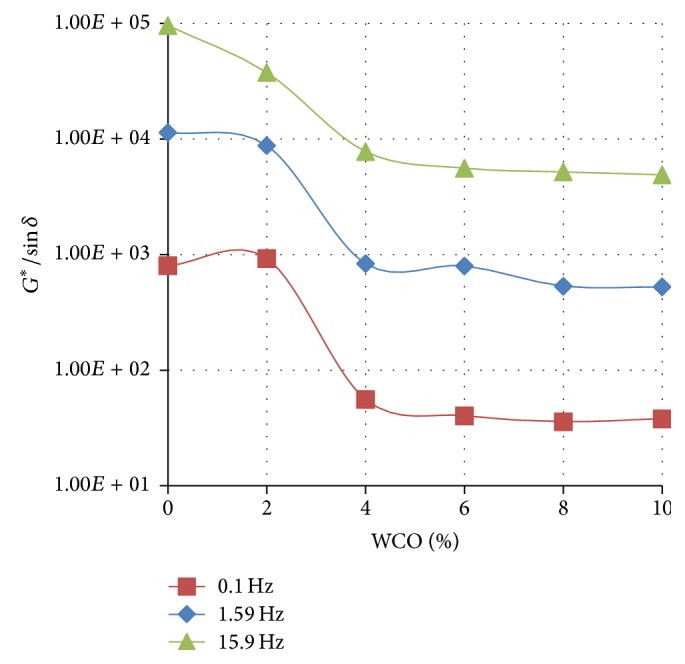
The variation of the rutting resistance parameter (*G* ^*∗*^/sin⁡*δ*) with increasing concentration of WCO in TLA : TPB (50 : 50) at various oscillating frequencies at 60°C.

Figures 4 [Fig fig5]–6 show the variation of the rutting resistance parameter (*G* ^*∗*^/sin⁡*δ*) with increasing concentration of WCO in TLA, TPB, and TLA : TPB (50 : 50) blend at three oscillating frequencies, respectively.

The results show that the rutting resistance of the parent binders was superior compared with their respective blends containing varying amounts of WCO. For all the three parent binders, as the concentration of the added WCO increases, the rutting resistance decreases (*G* ^*∗*^/sin⁡*δ* value decreases). As shown in Figure 4, the blends formulated using the TLA binder had the highest values of *G* ^*∗*^/sin⁡*δ* at the measured frequencies indicating that these blends will exhibit the highest rutting resistance of all the binders used. It is interesting to note that the values of *G* ^*∗*^/sin⁡*δ* at the measured frequencies for the TLA : TPB (50 : 50) blends were averaged between those of the blends containing TLA and TPB alone.

**Figure 7:**
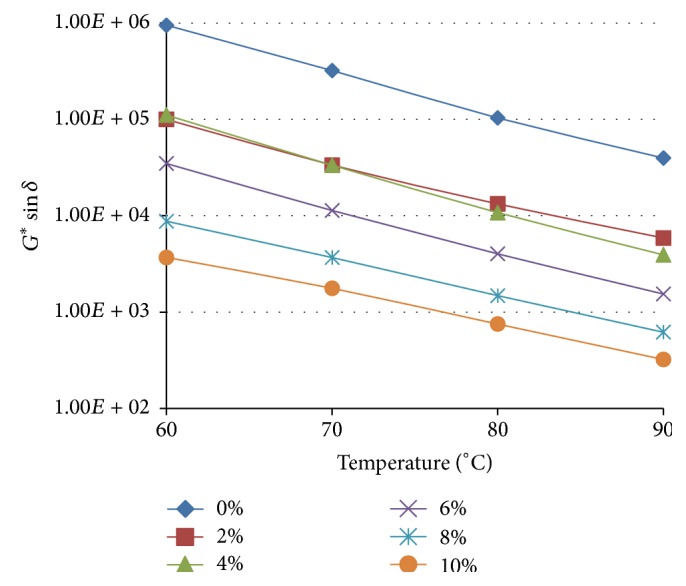
The variation of the fatigue cracking resistance parameter (*G* ^*∗*^sin⁡*δ*) of WCO modified TLA with increasing temperature at a frequency of 1.59 Hz.

The dependence of the fatigue cracking parameter (*G* ^*∗*^sin⁡*δ*) with the measuring temperature for the TLA asphaltic base binder and its WCO modified blends is shown in Figure 7.

**Figure 8:**
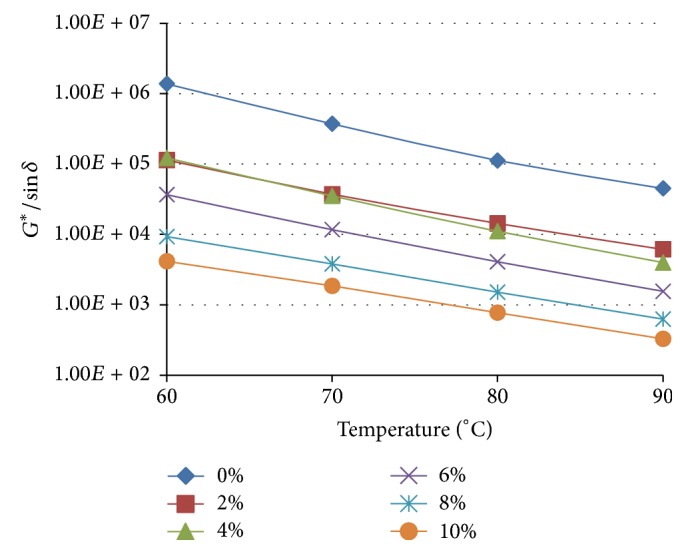
The variation of the rutting parameter (*G* ^*∗*^/sin⁡*δ*) of WCO modified TLA with increasing temperature at a frequency of 1.59 Hz.

The results demonstrate that the values of *G* ^*∗*^sin⁡*δ* for all the TLA blends gradually decreased as the measuring temperature was incrementally increased indicating that the fatigue cracking resistance increases as temperature increases. The dependence of the rutting parameter (*G* ^*∗*^/sin⁡*δ*) with the measuring temperature for TLA and its WCO modified blends is shown in Figure 8.

The values of *G* ^*∗*^/sin⁡*δ* for all the TLA blends gradually decreased as the measuring temperature was incrementally increased indicating that the rutting resistance decreases as temperature increases. The trends as shown in Figures 7 and 8 for the variation of the fatigue cracking parameter and the rutting resistance for TLA and its WCO modified blends were similar to what was observed for the WCO modified TPB and TLA : TPB (50 : 50) blends.

**Figure 9:**
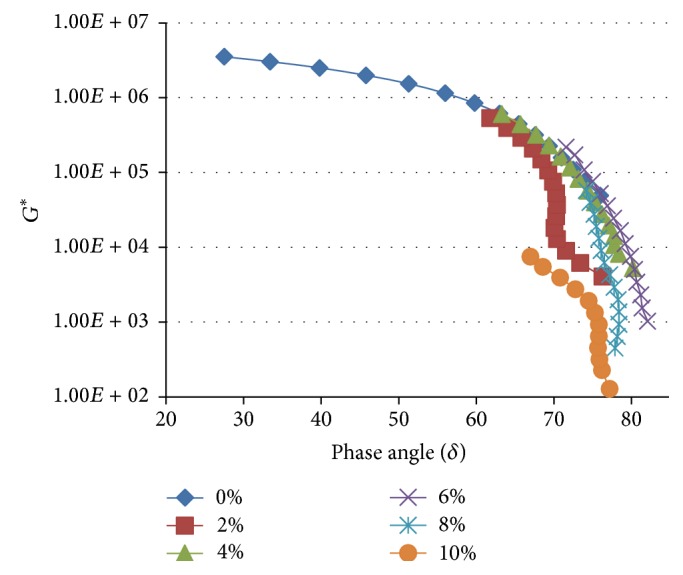
Black curves for WCO modified TLA blends measured at 1.59 Hz and 60°C.

**Figure 10 fig10:**
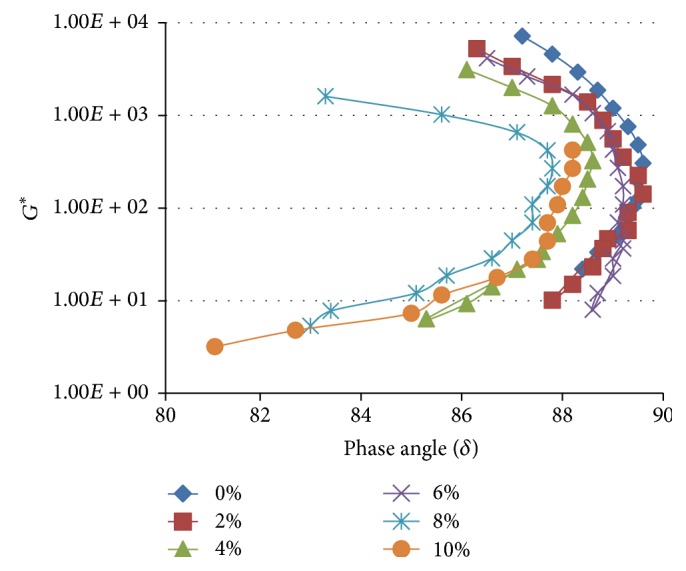
Black curves for WCO modified TPB blends measured at 1.59 Hz and 60°C.

**Figure 11:**
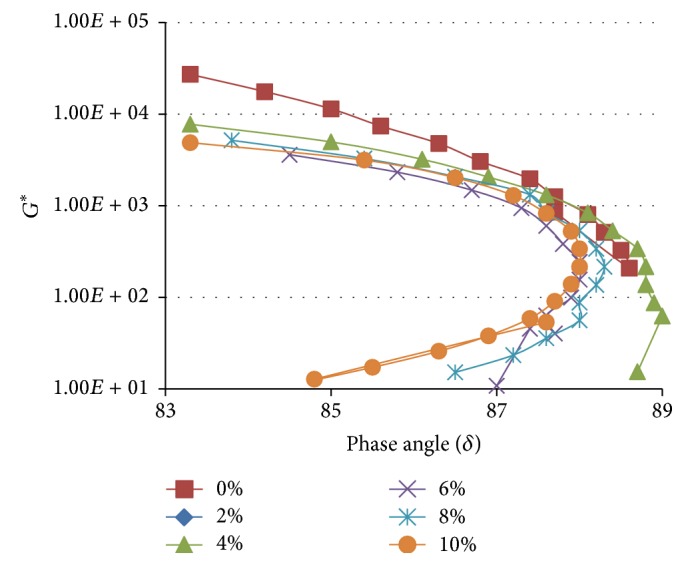
Black curves for WCO modified TLA : TPB (50 : 50) blends measured at 1.59 Hz and 60°C.

In addition to the analysis performed using the Strategic Highway Research Program presented above, the results can also be analysed utilizing the alternative rheology-performance relationship described by the Asphalt Research Program Superpave specification [10]. This approach recommends a high *G* ^*∗*^ (stiffness) but low *δ* (elastic) structure to reduce rutting and low values of *G* ^*∗*^ and *δ* to reduce the fatigue cracking. The graphical variation of *G* ^*∗*^ with *δ* is referred to as a black curve; that is, “a series of bitumens differing in penetration but not temperature susceptibility (penetration index) will give a single black curve” [20, 22]. The deviations from this base curve indicate changes in composition or structure caused by processing, ageing, or polymer addition. The black curves obtained in this study for the three asphaltic binders and their various WCO modified blends at a frequency of 1.59 Hz at a temperature of 60°C using the Asphalt Research Program Superpave specification are depicted in Figures 9 [Fig fig10]–11.

As shown in Figures 9–11, the addition of WCO to TLA, TPB, and TLA : TPB (50 : 50) blend generally resulted in the black curves shifting towards a slightly less stiff (slightly lower *G* ^*∗*^) and more elastic response (lower *δ*) compared to the curve of the parent asphalt in each case. This according to the Asphalt Research Program Superpave specification [10] should minimize fatigue cracking but increase susceptibility to rutting. The analytical findings using the Asphalt Research Program Superpave specification and the Strategic Highway Research Program produced exact conclusions thus validating the results of this study. The results of this study have also offered supporting evidence that the two approaches utilized to relate the rheological parameters (*G* ^*∗*^ and *δ*) and pavement performance attributes of rutting and fatigue cracking are complementary.

## 4. Conclusion

The addition of WCO to TLA, TPB, and TLA : TPB (50 : 50) blend resulted in changes in the rheological properties of the blends as demonstrated by changes in the phase angle, *δ* (elasticity), and the complex modulus, *G* ^*∗*^ (stiffness), of the blends. The results found that for the three Trinidad asphaltic binders we have the following:

(1) The rutting resistance of the parent binders was superior compared with their WCO modified. For all three parent binders, as the concentration of the added WCO increases, the rutting resistance decreases (*G* ^*∗*^/sin⁡*δ* value decreases).
(2) As the concentration of WCO increases, the fatigue cracking resistance increases (*G* ^*∗*^sin⁡*δ* value decreases). The blends formulated using the TPB binder had the lowest values of *G* ^*∗*^sin⁡*δ* indicating that these blends will exhibit the maximum fatigue cracking resistance.
(3) The values of *G* ^*∗*^sin⁡*δ* and *G* ^*∗*^/sin⁡*δ* for the TLA : TPB (50 : 50) blends were between those of the blends containing TLA and TPB as the base binder, an expected observation as the TLA : TPB (50 : 50) blends are mixtures of TLA and TPB.
(4) As temperature was incrementally increased, the value of *G* ^*∗*^sin⁡*δ* and *G* ^*∗*^/sin⁡*δ* incrementally decreased for the three asphaltic parent binders and all of the WCO modified asphaltic blends indicating an increase in the resistance to fatigue cracking and a decrease in the rutting resistance.
(5) This study demonstrated the capability to create customized asphalt-WCO blends to suit special applications and highlights the potential for WCO to be used as an environmentally attractive option for improving the use of Trinidad asphaltic materials such as TLA and TPB.

## Figures and Tables

**Table 1:** Concentrations of sample blends.

Asphalt blend	Mass of sample 6 g					
	TLA		TPB		TLA : TPB (50 : 50)	
% WCO required in blends	Actual mass of WCO added (g)	Actual % WCO	Actual mass of WCO added (g)	Actual % WCO	Actual mass of WCO added (g)	Actual % WCO
0.00	0.00	0.00	0.00	0.00	0.00	0.00
2.00	0.12	2.00	0.13	2.17	0.13	2.17
4.00	0.24	4.00	0.24	4.00	0.24	4.00
6.00	0.37	6.17	0.37	6.17	0.36	6.00
8.00	0.49	8.17	0.48	8.00	0.48	8.00
10.00	0.61	10.17	0.61	10.17	0.61	10.17
